# Linking spatially distributed neuronal activation overlap to the limits of perceptual discrimination in rodent primary somatosensory cortex

**DOI:** 10.3389/fncom.2026.1876230

**Published:** 2026-08-14

**Authors:** Madison Jiang, Joseph J. Pancrazio, Thomas J. Smith

**Affiliations:** 1 School of Behavioral and Brain Sciences, The University of Texas at Dallas, Richardson, TX, United States; 2 Department of Bioengineering, The University of Texas at Dallas, Richardson, TX, United States

**Keywords:** computational model, intracortical microstimulation, rodent, sensory discrimination, somatosensory cortex

## Abstract

**Introduction:**

Intracortical microstimulation (ICMS) of the primary somatosensory cortex can evoke localized tactile percepts, yet the spatial factors that influence perceptual discrimination remain poorly defined. In prior work, we showed that discrimination accuracy between behaviorally evaluated ICMS-evoked percepts declines as stimulation sites converge across cortical depths and adjacent cortical columns. Those results suggest that overlap in neuronal recruitment may constrain perceptual differentiation.

**Methods:**

Here, we combine simulated data from a biophysically realistic computational model of the somatosensory cortex with previously collected behavioral data from rats to quantify how overlap in ICMS-evoked activation volume relates to discrimination performance. Within the model, ICMS patterns investigated behaviorally were simulated, and activation volumes were estimated by fitting a range of 50–100% capture ellipsoids to the spatial distribution of activated somata. Overlap in activation volumes between pairs of ICMS patterns was then quantified using the intersection-over-union (IoU) metric.

**Results:**

Across both single- and four-shank microelectrode array configurations, we found that discrimination accuracy decreased in an exponential decay-like relationship (*R*^2^ = 0.88) as model-derived activation volume overlap increased. Independent of depth vs. lateral separation between ICMS pattern pairs, minimal overlap (IoU < 1%) was associated with high discrimination accuracy (>70%; average of 85%), whereas IoU values exceeding 20% corresponded to near-chance performance.

**Discussion:**

These results suggest that ICMS-evoked activation volume overlap between stimulation sites may provide mechanistic insight into the spatial limits of perceptual discrimination in ICMS applications. More broadly, these findings may help guide future investigations aimed at determining appropriate electrode spacing and stimulation strategies for sensory neuroprosthetic design.

## 1 Introduction

Intracortical microstimulation (ICMS) of the primary somatosensory cortex can evoke localized tactile percepts and is being explored as a tool for sensory restoration in brain–computer interface applications (Flesher et al., 2016; Fifer et al., 2022; Shelchkova et al., 2023). Both animal and human studies have shown that stimulation through implanted microelectrode arrays (MEAs) can generate behaviorally meaningful percepts (Flesher et al., 2016; Urdaneta et al., 2021; Greenspon et al., 2025). However, evoking a percept alone is not sufficient for functional feedback; brain–computer interfaces require stimulation patterns that can be reliably discriminated against to convey differences in spatial location or stimulus identity (Romo and Rossi-Pool, 2020). Although some studies have examined ICMS discrimination across electrode sites and stimulation parameters (Romo et al., 1998; Kim et al., 2015; Overstreet et al., 2016), the spatial resolution of ICMS (i.e., the minimum physical separation at which two ICMS patterns remain perceptually distinct) remains poorly defined (Smith et al., 2025). This uncertainty is compounded by the context that MEAs are typically designed for neural recording applications rather than electrical stimulation. Specifically, electrode spacing is often chosen without a clear understanding of how stimulation-evoked neural activation may constrain perceptual resolution (Erofeev et al., 2022; Yi et al., 2022). Physiological and modeling work has shown that ICMS activates axons near the electrode-site, producing antidromic activation of somata distributed throughout a cortical volume (Histed et al., 2009; Kumaravelu et al., 2022). Thus, stimulation at nearby sites may recruit overlapping neuronal populations and generate percepts that are difficult to distinguish between.

Our recent behavioral study in rat primary somatosensory cortex demonstrated that discrimination accuracy, based on behaviorally evaluated ICMS-evoked perceptual activity, decreases as stimulation sites converge across cortical depths and adjacent cortical columns (Smith et al., 2025). Experimental results support the notion that spatial overlap in activation volumes of recruited neurons may limit perceptual discriminability. This observation thus raises the question of how much volumetric overlap between ICMS-evoked activation volumes occurs as stimulation sites converge and how the extent of that overlap relates to perceptual discrimination. In the present study, we address these questions by combining comparable *in silico* data from a biophysically realistic model of the primary somatosensory cortex (Kumaravelu et al., 2022) with *in vivo* behavioral data collected from Smith et al. (2025). First, we use the computational model to simulate spatial activation data based on the same ICMS characteristics and patterns used experimentally. Then, we estimate activation volumes based on somatic recruitment and quantify the spatial overlap between ICMS pattern pairs. By evaluating the relationship between activation overlap to discrimination performance, this work offers insight into the spatial constraints of ICMS-evoked perceptual discrimination while generating experimentally testable hypotheses relevant to stimulation-first MEA design.

## 2 Materials and methods

### 2.1 Computational model of the somatosensory cortex

For this study, we employed a biophysically realistic computational model of a macaque somatosensory cortex originally developed by Kumaravelu et al. (2022). Internally, the model represents a cortical column with anatomically detailed neuronal morphologies spanning cortical layers I–VI and includes explicit representations of axonal projections that enable simulation of antidromic propagation following electrical stimulation. For all simulations, the modeled cortical volume measured 400 × 400 μm in the horizontal plane (*x*- and *y*-axes) and extended 2,000 μm vertically (z-axis). In total, there were 6,410 neurons in the model, which resulted in a neuronal density of approximately 20,000 neurons/mm^3^. In our previous work, we expanded the functionality of this model to simulate multi-electrode stimulation patterns corresponding to those used in previously reported behavioral discrimination experiments (Smith et al., 2025). The same modeling extensions were employed in the present study. All simulations were performed using the NEURON simulation environment, compiled from source and executed on The University of Texas at Dallas high-performance computing cluster, Ganymede, which provided 16,000 CPU cores and over 800 GPUs optimized for large-scale neural modeling.

### 2.2 Electrode configurations and ICMS patterns

**FIGURE 1:**
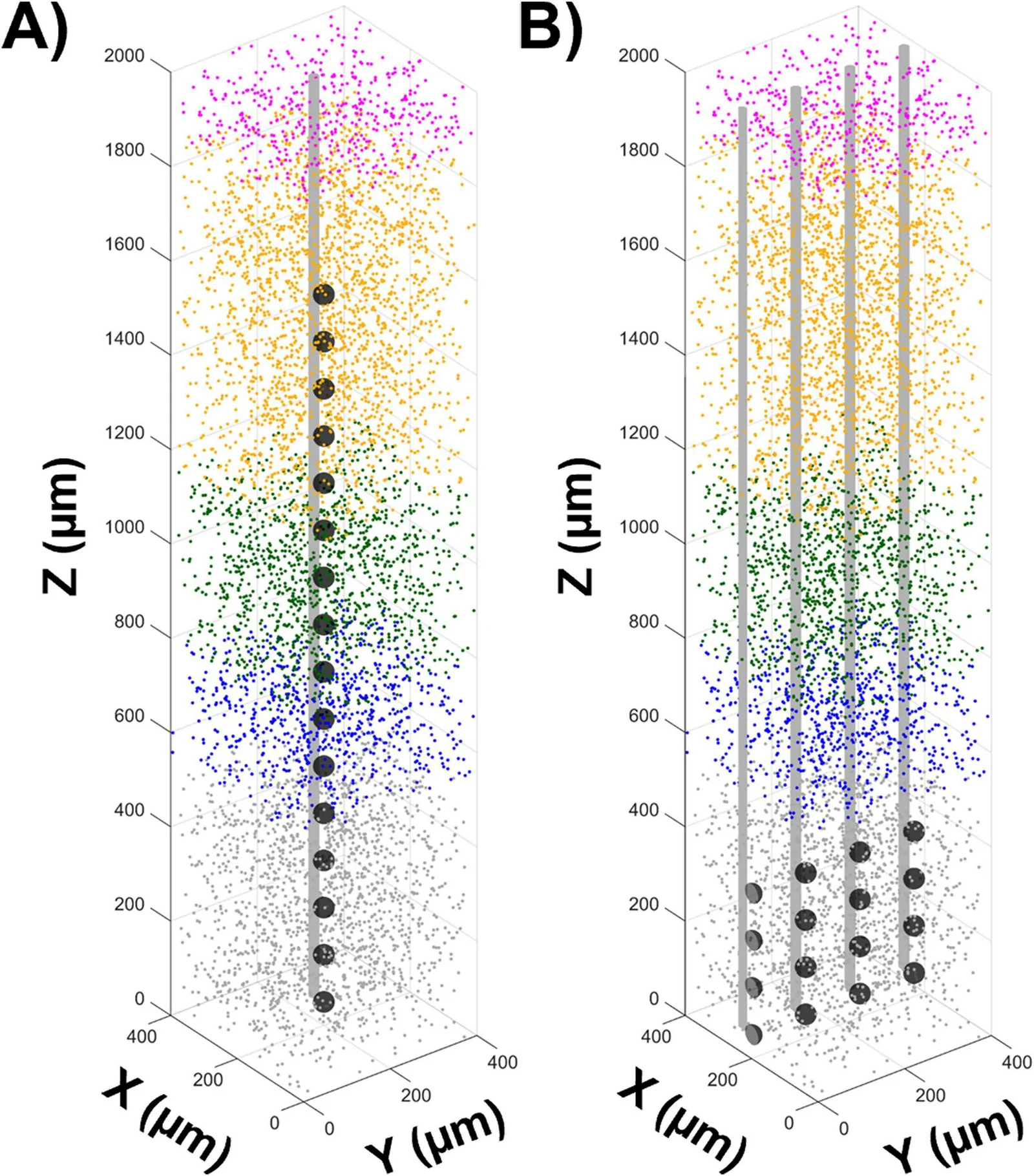
Spatial arrangement of modeled point sources within the simulated cortical column that correspond to implanted MEA electrode-site locations. **(A,B)** Point-source positions for the single-shank **(A)** and four-shank **(B)** array configurations. Electrode shanks (silver columns) and electrode sites (black spheres) are shown for visualization purposes only; stimulation was modeled exclusively at the point-source locations corresponding to each electrode site. Colored dots distributed throughout the column represent the individual soma locations of modeled neurons across cortical layers I–VI (pink = I, yellow = II/III, green = IV, blue = V, gray = VI).

ICMS was modeled as point source current injections, consistent with prior modeling studies (Overstreet et al., 2013, Kim et al., 2017). Point source locations matched the electrode-site positions of the implanted MEAs used during behavioral experiments (Smith et al., 2025). Behavioral experiments employed commercially available NeuroNexus silicon MEAs comprising 16 activated iridium electrode-sites (703 μm^2^ per site) in either a single- (A1 * 16-3 mm-100-703-CM16LP) or four-shank (A4 * 4-3 mm-100-125-703-CM16LP) configuration (see Figures 1A, B and Table 1). In both configurations, stimulation sites were positioned at model depths corresponding to the experimentally targeted MEA implantation tip-depth of 2 mm. The single-shank array consisted of 16 vertically aligned point sources spaced 100 μm apart along the z-axis (50–1550 μm; Figure 1A). The four-shank array consisted of a 4 × 4 arrangement of vertically aligned columns of point sources spaced 100 μm apart along the z-axis (50–350 μm) and laterally separated by a 125 μm pitch between adjacent columns (Figure 1B).

**TABLE 1:** XYZ coordinates of ICMS pattern point-source locations within the simulated cortical column. Coordinates correspond to the electrode-site positions used to model *in vivo* single-shank and four-shank stimulation patterns.

Single-shank ICMS patterns				Four-shank ICMS patterns			
Pattern #	X (μm)	Y (μm)	Z (μm)	Pattern #	X (μm)	Y (μm)	Z (μm)
S1	200	200	1250, 1350, 1450, 1550	F1	0	200	50, 150, 250, 350
S2	200	200	1050, 1150, 1250, 1350	F2	125	200	50, 150, 250, 350
S3	200	200	850, 950, 1050, 1150	F3	250	200	50, 150, 250, 350
S4	200	200	450, 550, 650, 750	F4	375	200	50, 150, 250, 350
S5	200	200	50, 150, 250, 350				

Nine ICMS patterns were defined identically to those described in Smith et al. (2025), with each pattern consisting of four simultaneously stimulated electrode sites (point sources). Briefly, five depth-dependent patterns were modeled for the single-shank configuration (S), while four laterally distinct patterns were modeled for the four-shank configuration (F). The spatial coordinates of all behaviorally-relevant ICMS pattern point sources are summarized in Table 1.

To supplement the original stimulation patterns and address spatial sampling limitations imposed by the MEA configurations and behavioral paradigm, seven additional ICMS patterns (shown in Table 2) were also simulated. For the single-shank configuration, patterns S6–S8 were defined at intermediate depths between existing stimulation sites. For the four-shank configuration, patterns F5–F8 were defined at intermediate lateral separations between adjacent shanks. However, we note that these additional patterns were only used in Section 3.2 to extend the range of spatial separations evaluated in the model when compared against activation volume overlap. The additional patterns were not included in behaviorally-relevant trend comparisons due to lack of corresponding discrimination accuracy data.

**TABLE 2:** XYZ coordinates of additional ICMS pattern point-source locations within the simulated cortical column.

Additional single-shank ICMS patterns				Additional four-shank ICMS patterns			
Pattern #	X (μm)	Y (μm)	Z (μm)	Pattern #	X (μm)	Y (μm)	Z (μm)
S6	200	200	1000, 1100, 1200, 1300	F5	150	200	50, 150, 250, 350
S7	200	200	950, 1050, 1150, 1250	F6	175	200	50, 150, 250, 350
S8	200	200	900, 1000, 1100, 1200	F7	200	200	50, 150, 250, 350
				F8	225	200	50, 150, 250, 350

### 2.3 Stimulation parameters

To characterize charge-dependent effects on activation volume and somatic recruitment, multiple simulations were conducted *in silico* using charge magnitudes ranging from 1.25 to 2.75 nC/ph per electrode in 0.25 nC/ph increments, reflecting the average range of detection thresholds reported for multi-channel stimulation in rats (Smith et al., 2023). At each point source, a single cathodic-leading biphasic pulse was delivered with a phase duration of 200 μs, an interphase interval of 40 μs, and a stimulation frequency of 320 Hz. A stimulation frequency of 320 Hz was selected to match the ICMS parameters used during the previously published behavioral discrimination experiments (Smith et al., 2025), thereby enabling a similar comparison between simulated activation overlap and behavioral performance. Each stimulation pattern consisted of the simultaneous activation of four point sources.

After establishing a robust and interpretable approach for estimating activation volume across various stimulation charge magnitudes and cortical depths (see sections 2.5 and 3.1), additional simulations were then completed using behaviorally derived charge magnitudes for each ICMS pattern to make direct comparisons with experimental results. All stimulation charge magnitudes used during these simulations were selected based on the average 75% perception threshold values determined behaviorally for each ICMS pattern (Smith et al., 2025). For the single-shank configuration, pattern-specific charge magnitudes varied with cortical depth to reflect the normalization procedures used during behavioral testing. Specifically, single-shank patterns S1–5 were simulated using charge magnitudes of 3.33, 2.36, 1.70, 1.41, and 1.39 nC/ph per electrode, respectively. For the four-shank configuration, each ICMS pattern was simulated using a charge magnitude of 2.40 nC/ph per electrode.

### 2.4 Identification of activated neurons

**FIGURE 2:**
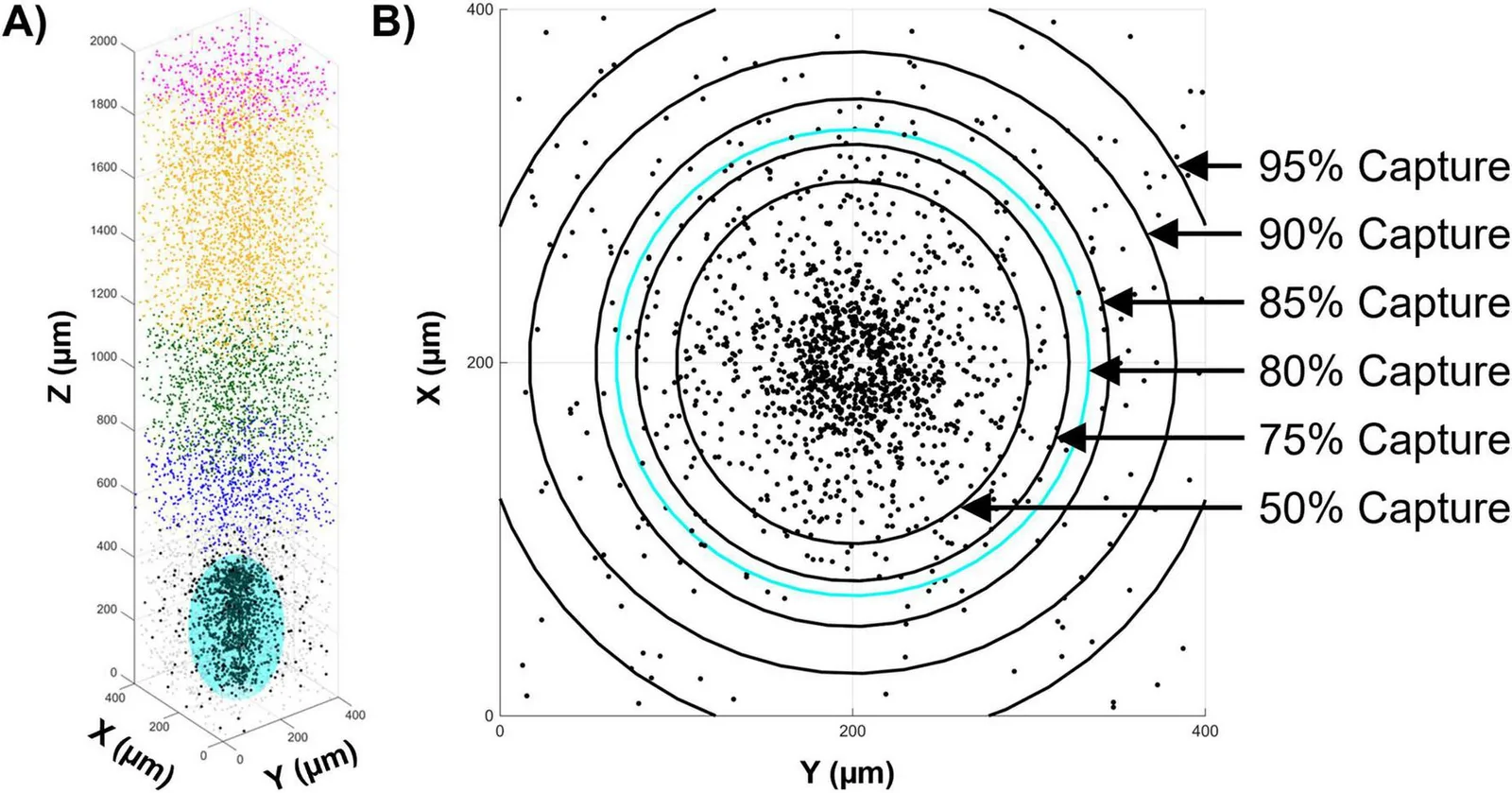
Example of ICMS-evoked somatic activation and its spatial representation across various ellipsoid capture thresholds. **(A)** Spatial distribution of activated somata following simulation of a centralized four-shank pattern (*x* = 200 μm, *y* = 200 μm; *z* = 50, 150, 250, 350 μm) at 2.40 nC/ph per electrode; black dots denote somatic activation, teal ellipsoid visualizes an 80% capture threshold. **(B)** Top-down view of the activated somata from panel **(A)**, illustrating the spatial distribution of neural recruitment in the x–y plane. Overlaid ellipsoids boundaries represent capture thresholds of 50, 75, 80, 85, 90, and 95% of activated somata at *z* = 200 μm.

To identify neurons directly activated by each ICMS pattern, simulations were run for 200 ms prior to stimulation and monitored for 500 μs following stimulus onset. This analysis window was chosen to isolate direct stimulation-evoked recruitment while minimizing contributions from synaptic transmission or network-level propagation (Smith et al., 2025). Membrane potentials of all axonal segments were recorded at 25 μs intervals. Axonal segments were classified as activated if their membrane potential exceeded -20 mV at any time during the analysis window. A neuron was considered activated if at least one of its axonal compartments met this criterion. Activated somata were subsequently identified through antidromic propagation from the site of axonal activation (Kumaravelu et al., 2022). An example distribution of activated somata for a centralized four-shank ICMS pattern (*x* = 200 μm, *y* = 200 μm; *z* = 50, 150, 250, 350 μm) is shown in Figure 2A. Due to consistent charge-dependent activation trends in layer VI of the model, this centralized pattern not only served as a proxy for all four-shank stimulation patterns, but also helped illustrate the spatial spread of stimulation-evoked recruitment across the x–y plane. Following each simulation, activation data were exported and analyzed using MATLAB (R2020a; MathWorks, Natick, MA, USA).

### 2.5 Estimation of activation volumes

To quantify the spatial distribution of neuronal recruitment evoked by each ICMS pattern, activation volumes were estimated by fitting a three-dimensional ellipsoid to the spatial coordinates of activated somata. The ellipsoid surface was defined using a standard ellipsoid equation:


$$\frac{{\left(x-x_{c}\right)}^{2}}{a^{2}}+\frac{{\left(y-y_{c}\right)}^{2}}{b^{2}}+\frac{{\left(z-z_{c}\right)}^{2}}{c^{2}}\leq 1$$ (1)

In Equation 1, (x_c_, y_c_, z_c_) defines the center of the ellipsoid along the x-, y-, and z-axes, while a, b, and c represent the semi-axis radii of the ellipsoid along those axes, respectively. Together, these parameters define the spatial extent of the activation volume. For each ICMS pattern in this study, the ellipsoid center was fixed at the centroid of the activated somata. Initial estimates of the semi-axis radii were then derived from the interquartile spatial distribution of activated somata along each axis. Specifically, the 75% interquartile span along each one-dimensional axis was used to define the initial semi-axis length values of a, b, and c . However, this step alone resulted in an ellipsoid that contained fewer somata than the capture threshold required.

Following initial estimates, the radii (*a*, *b*, and *c*) were then iteratively increased until a specified percentage of activated somata were enclosed within the ellipsoid volume, defined as the capture threshold. During each iteration, programmatic expansion proceeded in the following order: (1) *c* increased by 1 μm along the z-axis, (2) *b* increased by 0.3 μm along the x-axis, (3) *a* increased by 0.3 μm along the y-axis. After each proposed increment, the inclusion of additional somata within the ellipsoid was re-evaluated. The increment was retained only if it increased the captured fraction of activated somata. The algorithm terminated once the desired capture threshold was reached. If the target capture threshold could not be reached using these step sizes, a secondary expansion phase was applied using large increments (5 μm along the *z*-axis, 1 μm along the *x*-axis, and 1 μm along the *y*-axis) until the target threshold was satisfied. In the present study, capture thresholds ranging from 50 to 100% were evaluated to assess how the estimated activation volume changed as a function of the proportion of activated neurons included. Figure 2B illustrates the effect of different capture thresholds on the resulting ellipsoid size.

Finally, activation volumes were computed using a voxelized representation of the modeled cortical volume. Voxels located at coordinates (*x*, *y*, *z*) whose centers satisfied Equation 1 were classified as belonging to the activation volume (assigned a value of 1), while others were assigned a value of 0. Then, total activation volume was calculated by summing the number of included voxels and multiplying by the voxel volume. Each voxel volume measured 1 × 1 × 5 μm, consistent with the 1:1:5 aspect ratio of the modeled cortical column’s dimensions (400 × 400 × 2000 μm).

### 2.6 Quantification of activation volume overlap

To quantify the volumetric overlap between fitted ellipsoids of each ICMS stimulation pattern pair, we used the intersection-over-union (IoU) metric:


$$I\,o\,U=\frac{V_{o\,v\,e\,r\,l\,a\,p}}{V_{1}+V_{2}-V_{o\,v\,e\,r\,l\,a\,p}}\times 100\%$$ (2)

Here, *V*_1_ and *V_2_* denote the total volumes of the individual ellipsoids corresponding to each stimulation pattern, while *V*_overlap_ represents the intersecting (overlapping) volume between them. For each pair of stimulation patterns, activation volume overlap (*V*_*overlap*_) was computed using the voxelized cortical volume representations described in Section 2.5. Specifically, binary voxel arrays corresponding to each stimulation pattern’s activation volume ellipsoid set at a specified capture threshold (as defined by Equation 1) were combined using a logical AND operation to identify voxels within the volume of intersection. Similar to Section 2.5, the number of intersecting voxels was then summed and multiplied by the voxel volume to obtain *V*_*overlap*_. These values were subsequently used to compute IoU according to Equation 2.

**FIGURE 3:**
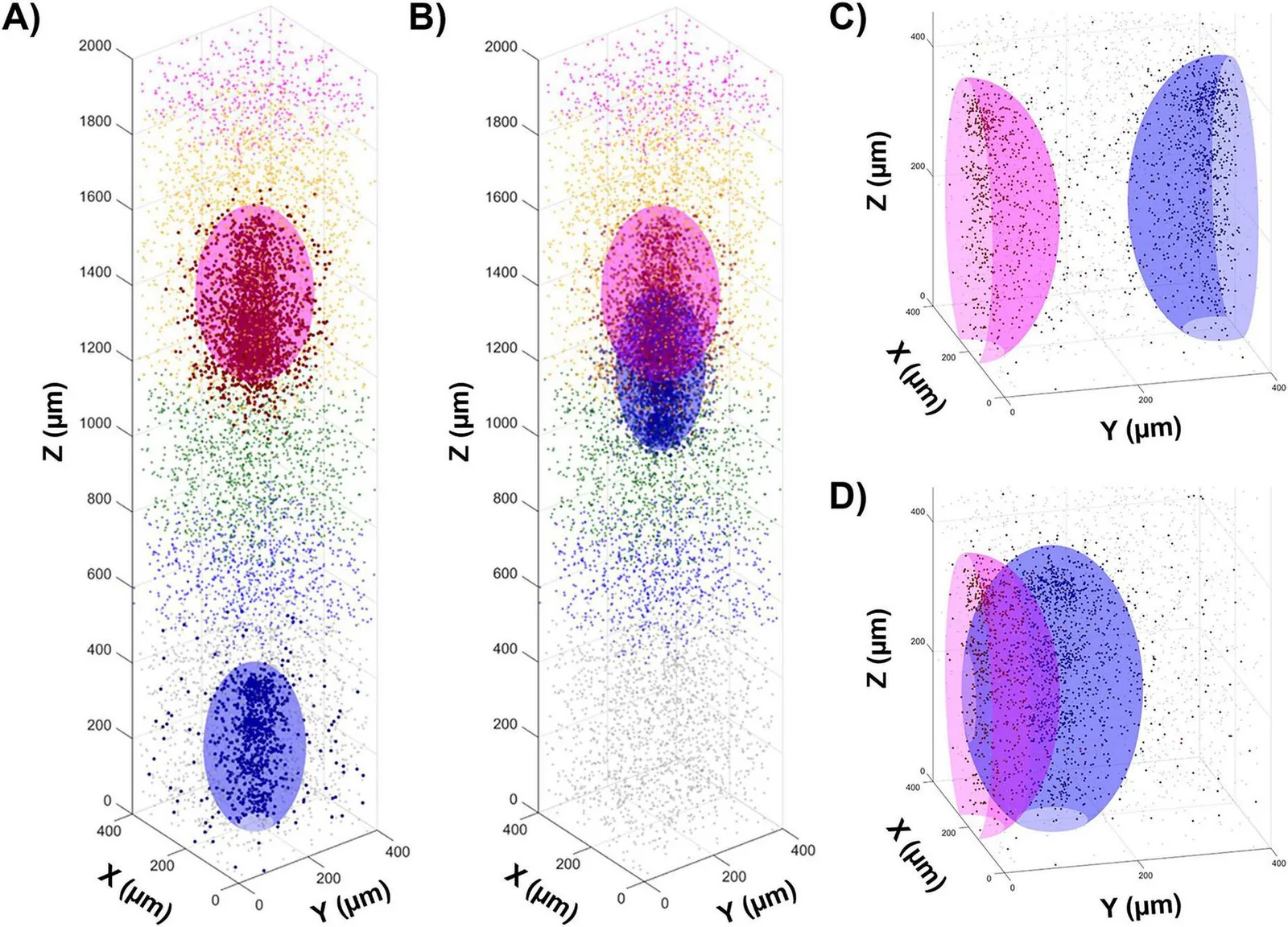
Examples of overlapping and non-overlapping activation volumes using an 80% ellipsoid capture threshold. **(A)** Non-overlapping activation volumes for pattern pair S1 vs. S5 of the single-shank configuration, simulated at 3.33 and 1.39 nC/ph per electrode, respectively. **(B)** Overlapping activation volumes for pattern pair S1 vs. S2 of the single-shank configuration (pattern S2 simulated at 2.36 nC/ph per electrode). **(C)** Non-overlapping activation volumes between shank 1 (pattern F1) and shank 4 (pattern F4) of the four-shank configuration, each simulated at 2.40 nC/ph per electrode. **(D)** Overlapping activation volumes between shank 1 and shank 2 (pattern F2) of the four-shank configuration at comparable charges to that described in **(C)**.

Although IoU is not widely applied in computational neuroscience, it is a well-established measure of spatial similarity used across multiple scientific domains where it is also referred to as the Jaccard Index (Bertels et al., 2019, Tang et al., 2021, Sheng et al., 2022). Because ICMS-evoked somatic activation tends to form spatially compact clusters (Stoney et al., 1968), IoU provides a scale-independent metric of shared neuronal recruitment between stimulation patterns that accounts for differences in activation volume size. Representative examples of overlapping and non-overlapping ICMS pattern pair ellipsoids for both array configurations are shown in Figure 3.

### 2.7 Behavioral data

Behavioral discrimination accuracy values used throughout this study were obtained from our previously published ICMS sensory discrimination experiments in rats (Smith et al., 2025). In that study, animals were trained to perform a two-choice sensory discrimination task in which they discriminated between pairs of ICMS stimulation patterns (one constant control pattern and one varying target pattern) by selecting the corresponding nose-poke response port. Correct identification of the presented ICMS pattern was rewarded with a sugar pellet. Behavioral comparisons were performed using pattern-specific stimulation amplitudes normalized to each pattern’s behavioral 75% perception threshold to account for differences in detection sensitivity across stimulation locations. Overall, the study’s dataset included seven unique behaviorally tested ICMS pattern pairs together with two pattern pair controls (S1 vs. S1 and F1 vs. F1), which represent complete activation overlap (IoU = 100%) by definition.

Behavioral experiments were initially performed in eight rats (four implanted with single-shank MEAs and four implanted with four-shank MEAs). Two animals were excluded because of loss of sensitivity to the control stimulation pattern, leaving three animals per array configuration for analysis. Across all experimental sessions, behavioral discrimination accuracy was calculated from approximately 685 trials per pattern pair for the single-shank group (3,423 total trials) and 747 trials per pattern pair for the four-shank group (2,987 total trials). Average discrimination accuracy values (mean ± SEM) for each stimulation pattern pair were then used for the present modeling analyses.

### 2.8 Statistical analysis

Behavioral discrimination accuracy values were obtained from previously published two-choice ICMS sensory discrimination experiments (Smith et al., 2025). The relationship between IoU values and discrimination accuracy scores across both single- and four-shank ICMS pattern pairs (*n* = 9) was evaluated by combining their datasets and fitting an exponential one-phase decay curve using GraphPad Prism 10 (v10.6.1; GraphPad Software, Boston, MA, USA). Here, the lower asymptote (plateau) was constrained to 50% to represent chance discrimination performance. For comparison with a simpler geometric descriptor, we also fit a one-phase association curve to the relationship between pattern pair centroid distance and discrimination accuracy. Here, the upper asymptote was constrained to 100% to represent perfect discrimination performance. For both models, R^2^ values were reported as indicators of association strength. Model robustness was assessed by calculating 95% confidence intervals for the fitted models and performing a leave-one-out sensitivity analysis, where each behavioral comparison was excluded individually before refitting. Both exponential models were selected based on previous behavioral results, demonstrating that discrimination accuracy decreases as stimulation sites spatially converge. As electrode sites become closer together, the resulting ICMS-evoked activation volumes are expected to exhibit greater overlap, resulting in percepts that are progressively more difficult to discriminate between until reaching near-chance performance.

## 3 Results

In this study, we examined how spatial overlap between ICMS-evoked activation volumes relates to perceptual discrimination performance. Using a biophysically realistic model of the somatosensory cortex, we first characterized how stimulation charge influences activation volume estimates derived from the spatial distribution of activated somata. We then quantified overlap between activation volumes across various stimulation patterns using the IoU metric and compared these values against average behavioral discrimination accuracy scores previously reported in rats (Smith et al., 2025).

### 3.1 Charge-dependent scaling effects and geometric characterizations of activation volumes

**FIGURE 4:**
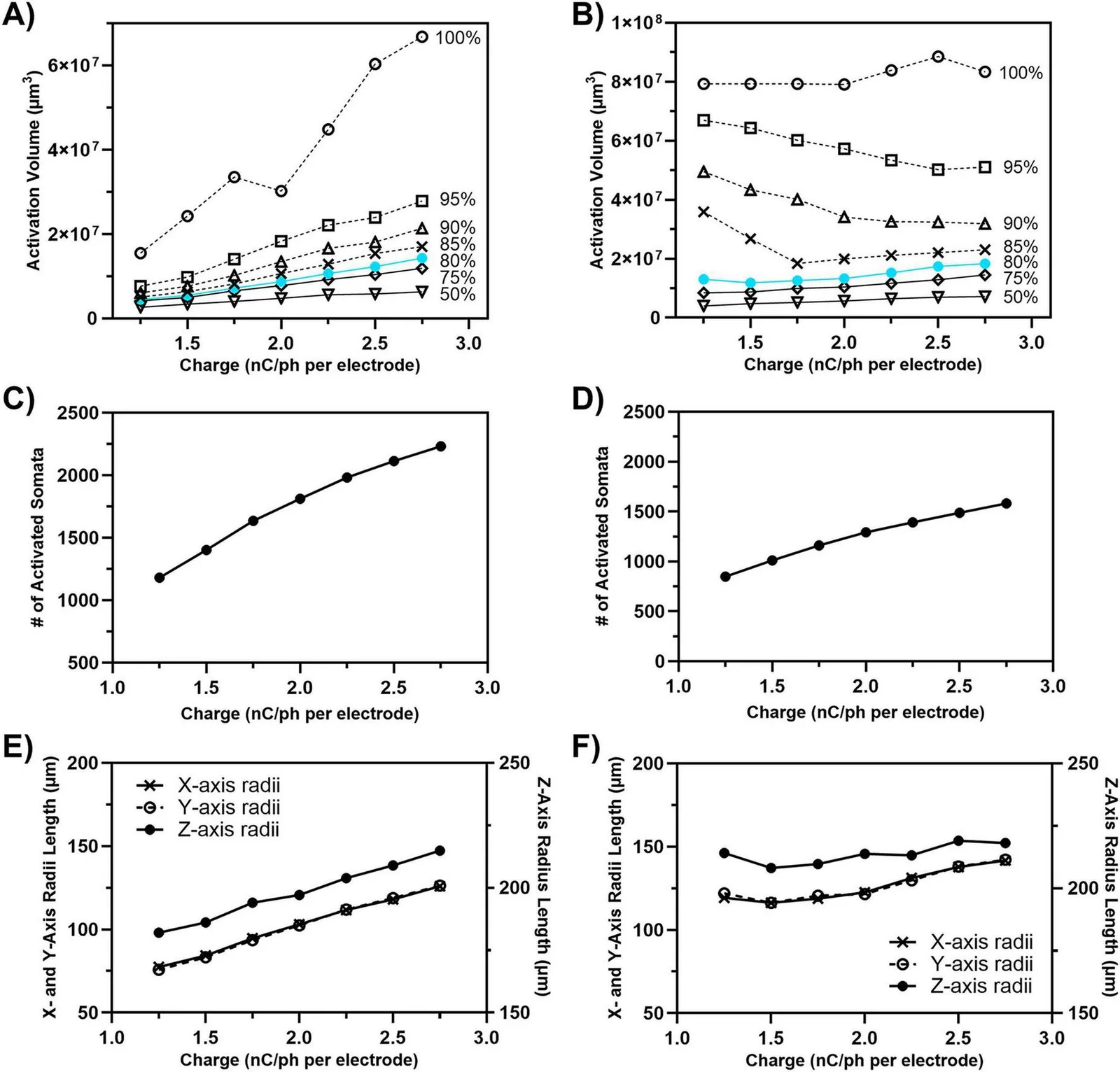
Relationship between ICMS charge magnitude, activation volume, somatic recruitment, and ellipsoid geometry. **(A,B)** Activation volume as a function of stimulation charge magnitude across ellipsoid capture thresholds (50–100%) for representative single-shank patterns S1 [**(A)**; superficial] and S5 [**(B)**; deep]. **(C,D)** Number activated somata as a function of stimulation charge for patterns S1 **(C)** and S5 **(D)**. **(E,F)** Charge-dependent changes in ellipsoid semi-axis radii for the 80% capture threshold in relation to pattern S1 **(E)** and S5 **(F)**.

To establish a robust and interpretable measure of spatial recruitment, we first evaluated how activation volume estimates depend on stimulation charge and the capture threshold percentage chosen for the ellipsoid fit. Capture thresholds ranging from 50 to 100% were assessed across stimulation charge magnitudes of 1.25–2.75 nC/ph per electrode. To account for differences in cortical depth, two representative stimulation patterns were examined in Figure 4: single-shank pattern S1 (Figure 4A), corresponding to a superficial stimulation location, and single-shank pattern S5 (Figure 4B), corresponding to a deeper stimulation location that is also spatially equivalent to the centralized four-shank stimulation pattern proxy described in Figure 2. These patterns represent the most vertically separated stimulation sites within the single-shank array and therefore provide a useful comparison for assessing capture threshold stability across both stimulation charge magnitude and cortical depth.

Across our range of capture thresholds, activation volume generally increased with stimulation charge for both patterns. However, for the deeper pattern S5, capture thresholds above 80% exhibited non-monotonic behavior, with some activation volume estimates appearing larger at lower charge magnitudes than at higher ones. For instance, at an 85% capture threshold, pattern S5 exhibited a local minimum in activation volume at 1.76 nC/ph per electrode. As charge magnitude increased to 1.75 nC/ph per electrode, activation volume decreased from 3.6 × 10^7^ to 1.8 × 10^7^ μm^3^. Beyond 1.75 nC/ph per electrode, activation volume increased monotonically, reaching a maximum of only 2.3 × 10^7^ μm^3^. Notably, these higher charge magnitude trends were consistent with those observed for S5 at capture thresholds of 50, 75, and 80%, as well as with most trends in pattern S1 across all charge magnitudes. Together, these findings indicate that the ellipsoid expanded disproportionately at lower charge magnitudes to capture sparsely distributed somata.

To support this argument, we also examined the total number of activated somata within the entire cortical column as a function of charge magnitude. For both ICMS patterns, we found that the number of activated somata increased consistently with stimulation charge (Figures 4C, D). This contrast shows that decreases in activation volume using larger capture thresholds are geometric artifacts of the ellipsoid fitting procedure rather than underlying changes in neuronal recruitment. Based on these observations, an 80% ellipsoid capture threshold was selected for all subsequent analyses, representing the highest capture percentage that yielded relatively stable, monotonic increases in activation volume across all cortical depths and ICMS patterns, while minimizing artifacts associated with sparse neuronal recruitment.

Supplementary Figure 1 extends analysis for patterns S2, S3, and S4 by examining the total number of activated somata, activation volume, and ellipsoid density as a function of charge magnitude for an ellipsoid volume calculated at an 80% capture threshold. While both activated somata and activation volume increased consistently with charge, activation density decreased as charge increased. This suggests that, within the scope of the model, activation volume increases faster than somatic recruitment.

Utilizing the 80% capture threshold, we next characterized how stimulation charge shapes the geometry of the resulting activation volumes. As shown in Figures 4E, F, increasing stimulation charge magnitude resulted in gradual expansion of the fitted ellipsoids along all spatial axes. The *x*- and *y*-axis radii increased from 116 to 142 μm, while the z-axis radius ranged from 208 to 219 μm across the tested range. Notably, expansion along the z-axis was consistently greater than in the lateral dimensions, resulting in anisotropic, elongated activation volumes. This asymmetry reflects the vertical arrangement of the four stimulated electrode sites, which produces a greater spread of activation along cortical depth than laterally. As a result, activation volumes were not spherical. Thus, the degree of overlap between activation volumes of stimulation patterns may be dependent on the direction of spatial separation (i.e., vertical pattern separation required larger centroid distances than lateral separations to avoid activation overlap). Supplementary Figures 1, 2 confirm that these scaling relationships are consistent across stimulation patterns and cortical depths when applying the 80% capture threshold, with Supplementary Figure 2 extending the geometric analysis to intermediate stimulation patterns (S2–S4). Together, these results establish a geometric framework for interpreting activation overlap and its relationship to perceptual discrimination in the next section.

### 3.2 The relationships between distance, IoU, and discrimination accuracy

**FIGURE 5:**
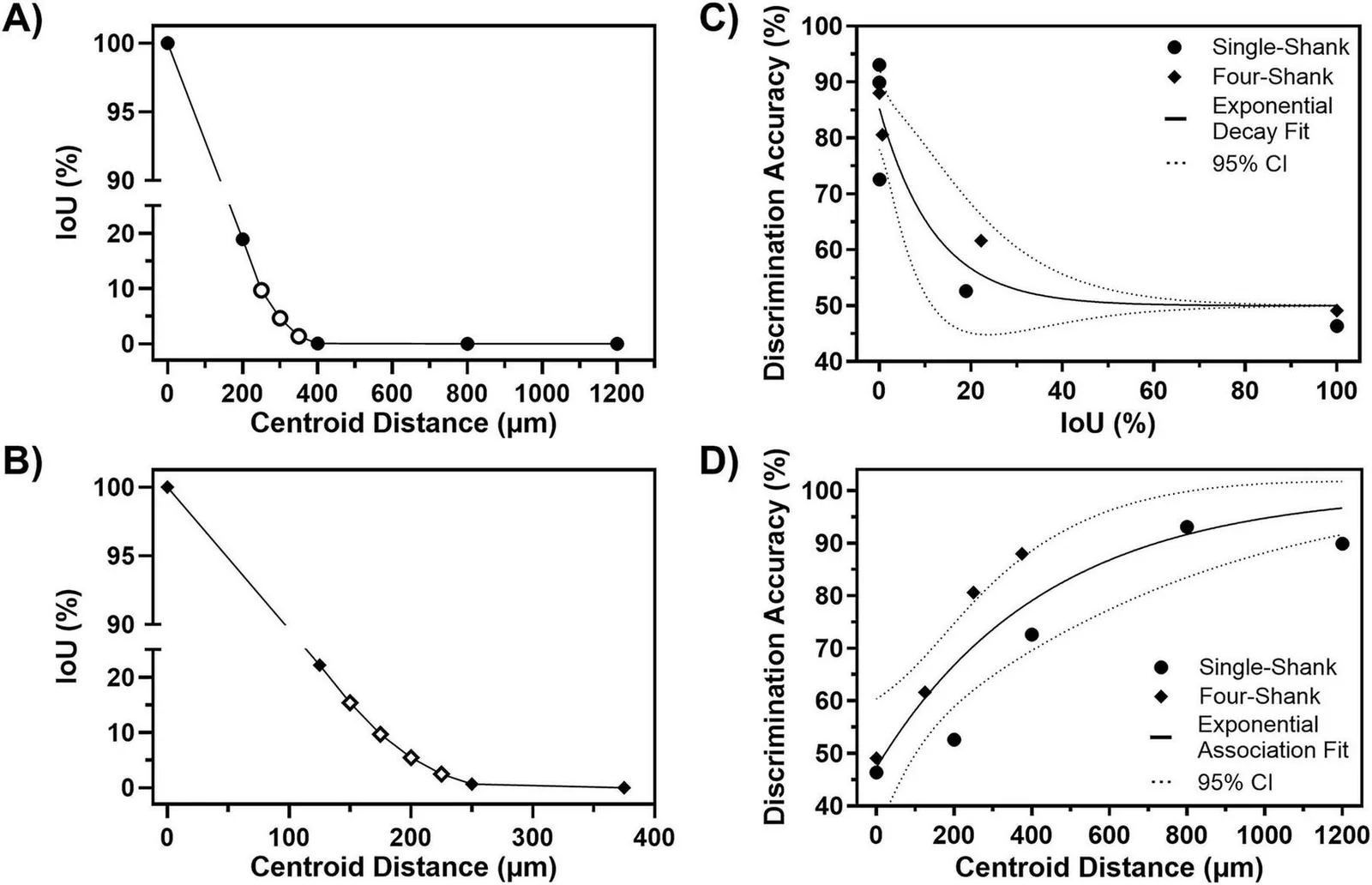
Relationship between spatial separation, activation overlap, and behavioral discrimination. **(A,B)** IoU as a function of vertical [**(A)**; single-shank] and lateral [**(B)**; four-shank] separation between stimulation pattern pairs. Black markers denote pattern pairs with corresponding behavioral data, while white markers represent additional simulated comparisons. **(C)** Relationship between IoU and behavioral discrimination accuracy across both MEA configurations. Data points from single- and four-shank configurations are combined, demonstrating a consistent exponential decay relationship between activation overlap and perceptual discriminability. The fitted exponential decay curve (*R*^2^ = 0.88) and 95% confidence intervals are shown. **(D)** Relationship between centroid distance and behavioral discrimination accuracy across both MEA configurations. Data points are fit with a one-phase exponential association model (R^2^ = 0.82); 95% confidence intervals are shown.

To determine how spatial overlap between activation volumes relates to perceptual discrimination, we first quantified IoU values across the centroid distances of 16 unique stimulation pattern pairs spanning both behaviorally tested (Table 1) and additional simulated conditions (Table 2). Afterward, the subset of patterns corresponding to *in vivo* experiments (*n* = 9) was plotted against their associated average behavioral discrimination accuracy score to evaluate their relationship. For both MEA configurations, we found that model-derived calculations of IoU decreased systematically with greater spatial distances between stimulation pattern pairs (Figure 5).

In the single-shank configuration (Figure 5A), pattern pairs separated by vertical distances greater than 400 μm (S1 vs. S3, S4, and S5) exhibited minimal IoU overlap (0.0–0.1%). In contrast, pattern pair S1 vs. S2, which shared two electrode-sites and differed in depth by only 200 μm, exhibited a substantially higher IoU overlap percentage of 18.9%. As expected, the control comparison S1 vs. S1 (0 μm separation) corresponded to complete activation overlap (IoU = 100%). A similar relationship was observed in the four-shank configuration (Figure 5B), but with a more rapid decline in overlap across lateral distances. Stimulation patterns on adjacent shanks (pattern pair F1 vs. F2; 125 μm separation) exhibited an IoU overlap value of 22.2%. Subsequently, pattern pairs F1 vs. F3 and F4 (250 and 375 μm separations) displayed a sizable reduction in IoU value, yielding minimal (0.6%) to no overlap (0.0%), respectively. The control comparison F1 vs. F1 (0 μm separation) corresponded to complete activation overlap (IoU = 100%). Notably, IoU overlap reached a value of zero at shorter distances in the four-shank configuration than in the single-shank configuration, reflecting the anisotropic geometry of activation volumes.

When relating IoU to behavioral performance, an exponential decay-like trend emerged across both configurations that was independent of cortical depth vs. lateral pattern separations (Figure 5C). Here, higher IoU volumetric overlaps exceeding ∼20% were associated with near-chance discrimination accuracy scores, whereas minimal overlap (IoU < 1%) corresponded to accuracy scores > 70% with an average of 85%. This trend was consistent across both configurations: in the single-shank condition, pattern pairs with minimal overlap (S1 vs. S3, S4, and S5) exhibited high average ± SEM discrimination accuracy scores (72.6 ± 2.5%, 93.1 ± 0.9%, and 89.9 ± 1.6%, respectively), while the pair S1 vs. S2, which shared an IoU overlap value of 18.9%, resulted in near-chance performance at an average ± SEM score of 52.6 ± 1.8%. Similarly, in the four-shank configuration, pattern pairs with minimal overlap (F1 vs. F3 and F4) were associated with high average ± SEM discrimination accuracy scores (80.6 ± 3.0% and 88.6 ± 1.9%), whereas the pair with the greatest overlap (F1 vs. F2; IoU = 22.2%) resulted in a reduced score of 61.6 ± 2.7%. The control comparisons S1 vs. S1 and F1 vs. F1 (IoU = 100%) yielded similar average ± SEM discrimination accuracy scores of 46.4 ± 2.1% and 49.1 ± 1.9%, respectively. When plotting data from both configurations, the overall relationship was well described by an exponential decay function (Figure 5C; *Y* = 35.3*e*^−0.1*X*^ + 50.0, *R*^2^ = 0.88), reflecting the saturating nature of behavioral performance at both low and high IoU values. Moreover, the fitted model remained robust to leave-one-out sensitivity analysis (R^2^ = 0.85–0.95), and corresponding 95% confidence intervals are shown in Figure 5C. To further evaluate the sensitivity of the observed relationship to the selected ellipsoid capture threshold, IoU values were recalculated using capture thresholds of 75%, 85%, and 90%. Across all thresholds evaluated, the relationship between IoU and behavioral discrimination remained highly consistent (R^2^ = 0.88–0.89), with comparable leave-one-out sensitivity analysis results (R^2^ = 0.85–0.96; Supplementary Table 1).

To compare activation overlap with a simpler geometric descriptor, centroid distance between stimulation pattern pairs was also evaluated in relation to behavioral discrimination (Figure 5D). Similar to IoU, discrimination accuracy increased with centroid distance and was reasonably described by a one-phase exponential association model (Figure 5D; *Y* = 100.0−52.7*e*^−0.002*X*^, *R*^2^ = 0.82). The fitted relationship also remained robust to leave-one-out sensitivity analysis (R^2^ = 0.76–0.89), and 95% confidence intervals are shown in Figure 5D. Although centroid distance exhibited a very similar relationship with behavioral discrimination, activation volume overlap demonstrated a slightly stronger association (Figure 5C; R^2^ = 0.88 vs. Figure 5D; R^2^ = 0.82) and a lower residual standard deviation (Sy.x = 6.84 vs. 8.49).

Overall, these results suggest that ICMS-evoked activation volume overlap between stimulation sites may provide mechanistic insight into the spatial limits of perceptual discrimination in ICMS applications, with greater overlap associated with reduced discrimination performance.

## 4 Discussion

In this study, we combined a biophysically realistic computational model of the somatosensory cortex with previously reported behavioral data to quantify how spatial overlap of ICMS-evoked neuronal activation relates to perceptual discrimination. By estimating activation volumes from somatic recruitment and computing their overlap using the IoU metric, we identified a relationship between model-derived activation overlap and behavioral discrimination performance across stimulation patterns that varied in cortical depth and lateral separation.

Overall, our results suggest that activation overlap is strongly associated with perceptual discriminability. Consistent with prior behavioral findings showing reduced discrimination accuracy as stimulation sites converge (Smith et al., 2025), we observed that pattern pairs with minimal overlap (IoU < 1%) were associated with high discrimination accuracy, whereas overlap values exceeding ∼20% corresponded to near-chance performance. This relationship was well described by an exponential decay model, consistent with the interpretation that perceptual separability may degrade as shared neuronal recruitment increases. Although centroid distance also exhibited a strong relationship with behavioral discrimination, activation volume overlap demonstrated a slightly stronger association that more directly reflects the spatial extent and geometry of stimulation-evoked neuronal recruitment. More importantly, these relationships were preserved across both depth-dependent and lateral stimulation configurations despite differences in charge magnitude and activation volume size. Furthermore, these results suggest that the limits of perceptual discrimination are not entirely constrained by the direct size of activation volumes, but at least partially defined by the degree to which those volumes overlap. From the modeling results, we showed that increases in stimulation charge magnitude also expands activation volume (Figure 4), which may increase the likelihood of overlap in neural recruitment between nearby sources. Consequently, future ICMS studies may need to consider how electrode spacing interacts with stimulation intensity to preserve perceptual separability.

Building on this relationship between activation overlap and perceptual discriminability, these findings provide a baseline quantitative modeling framework for informing the design of microelectrode arrays (MEAs) intended for stimulation-based applications. Many commonly used arrays, such as Michigan-style devices, are developed for neural recording and do not account for the spatial spread of stimulation-evoked activation (Delhaye et al., 2016; Yi et al., 2022). In contrast, our results suggest that electrode spacing should be explicitly defined relative to the spatial extent of neuronal activation at behaviorally relevant stimulation charge magnitudes, rather than inherited from recording-oriented designs. As an illustrative example, our prior behavioral work (Smith et al., 2025) targeted a discrimination accuracy of approximately 75%. Based on the relationship shown in Figure 5C, achieving this level of performance corresponds to an IoU activation overlap of approximately 1%. In the present model, this IoU overlap level is associated with an electrode separation of approximately 360 μm along the cortical depth axis (Figure 5A) or 240 μm across adjacent cortical columns (Figure 5B). While these values are model- and parameter-specific, they demonstrate how activation overlap could be used to inform electrode spacing in a principled manner. However, when wide electrode spacing is not feasible due to anatomical or surgical constraints (Delhaye et al., 2016), alternative stimulation strategies such as current steering may help preserve perceptual separability. By distributing current across multiple electrodes, current steering can effectively shift activation patterns within the cortex while maintaining separation between physical electrode sites (Chen et al., 2021; Kunigk et al., 2022). The framework presented here provides a quantitative basis for evaluating such approaches by relating stimulation parameters to predicted activation overlap and known behavioral outcomes.

Notably, the present findings highlight spatial overlap as a key factor of perceptual similarity under fixed stimulation parameters, including the 320 Hz stimulation frequency used throughout this study. However, prior work has shown that temporal features such as stimulation frequency can independently modulate perceptual quality even when delivered through the same electrode (Callier et al., 2020). In that study, multiple discriminable percepts were reported across a frequency range of approximately 10–200 Hz at constant amplitude. This suggests that temporal modulation may influence perceptual differentiation with overlapping spatial recruitment, although not explored here. Taken together, these findings indicate that while spatial overlap constrains perceptual discriminability under fixed stimulation parameters, temporal features such as frequency may provide an additional axis for shaping perceptual distinctions beyond spatial resolution alone.

While present study relates spatial activation overlap with perceptual discrimination, several limitations should be considered when interpreting these findings. First, the computational model represents a simplified cortical volume that does not incorporate long-range network interactions or synaptic dynamics, which may influence the spatial and temporal spread of activation. Second, electrodes were modeled as idealized point sources, omitting effects of electrode geometry, impedance, and tissue–electrode interface properties. Third, the number of activated somata was used as a proxy for neuronal recruitment; however, the model’s neuronal density (∼20,000 neurons/mm^3^) is lower than that of biological tissue (Kumaravelu et al., 2022), and therefore absolute recruitment levels may be underestimated. Despite these limitations, the underlying cortical framework remains relevant. Although the model was developed using macaque cortical morphologies, it provides a useful approximation for the present analysis. Macaque and rodent somatosensory cortices share conserved features relevant to ICMS activation, including laminar organization and cortico-cortical connectivity, despite differences in laminar thickness (Harris and Shepherd, 2015). Moreover, prior studies have demonstrated that ICMS preferentially activates axons near the electrode and produces antidromic somatic activation across species (Histed et al., 2009; Yun et al., 2023). In the absence of rodent-specific models with comparable morphological detail, the macaque-based model provides a practical starting point for investigating whether activation overlap and behavioral discrimination covary. Importantly, the cortical depth coordinates used throughout this study should not be interpreted as strict anatomical correspondences between macaque and rat cortex. Rather, they provide a spatial frame of reference that preserves the relative separation between stimulation patterns used during the behavioral experiments, while also enabling comparisons of activation overlap within a computational model. However, the absolute spacing estimates reported in this study may vary across species because of differences in cortical morphology, neuronal density, and cortical thickness. Accordingly, the quantitative spacing values reported here should be interpreted as model-dependent estimates rather than species-specific design recommendations.

Future work should extend this framework to stimulation parameters and electrode designs commonly used in other ICMS studies. Incorporating larger cortical volumes, synaptic transmission, and network-level dynamics may further clarify how activation overlap evolves over time and how it constrains perceptual resolution in more complex neural systems. Future studies should also examine whether cortical laminae differ intrinsically in their contribution to perceptual discrimination. Although stimulation charge was normalized to each ICMS pattern’s behavioral perception threshold, this normalization does not rule out the possibility that layer-specific differences in cellular composition, connectivity, or projection targets contribute independently to perceptual discriminability. Another important extension of this work would be to evaluate more complex three-dimensional stimulation configurations that combine variations in both cortical depth and lateral electrode position, including diagonal separations between stimulation patterns. The present study examined depth-dependent and lateral stimulation patterns independently to enable direct comparison with the available behavioral discrimination data. As additional behavioral datasets become available, future studies could use the same activation-overlap framework to investigate whether the relationship between activation volume overlap and perceptual discrimination observed here extends to these more complex three-dimensional stimulation geometries. Such studies would help determine whether activation overlap remains a useful predictor of perceptual discrimination across increasingly complex stimulation geometries.

## 5 Conclusion

In summary, this study suggests that spatial overlap in ICMS-evoked neuronal activation may help explain the perceptual discrimination performance observed in intracortical stimulation paradigms. By comparing activation volume overlap, measured using the IoU metric, with behavioral performance across multiple stimulation configurations, we establish a framework for evaluating how spatial recruitment of neural populations constrains perceptual resolution. Importantly, our basic findings suggest that perceptual discriminability is governed not by the size of activation volumes alone, but by the degree to which those volumes overlap. This distinction contributes to a more principled understanding of how stimulation-evoked neuronal recruitment shapes perception and highlights the role of shared activation in limiting sensory differentiation. Together, these model-based results provide quantitative guidance for the design of stimulation-first MEAs and may inform the development of ICMS strategies aimed at maximizing perceptual separability in future sensory neuroprosthetic applications.

## Data Availability

The datasets presented in this study can be found in online repositories. The names of the repository/repositories and accession number(s) can be found below: https://github.com/msj220001/Ssctx-column-model-4-electrode-ICMS.
