# Supplementary material for: Linking spatially distributed neuronal activation overlap to the limits of perceptual discrimination in rodent primary somatosensory cortex
Source: Front Comput Neurosci. 2026 Aug 14;20:1876230. doi: 10.3389/fncom.2026.1876230 (PMC13522154; doi:10.3389/fncom.2026.1876230)
Supplement: Supplementary file 1 [file Data_Sheet_1.docx]

Supplementary Material

## 1.1 Supplementary Figures

**Supplementary Figure 1.** Charge-dependent activation volume, somatic recruitment, and ellipsoid density for patterns S2–S4. (A, C, E) Relationship between stimulation charge magnitude, activation volume (80% capture threshold), and number of activated somata for patterns S2 (A), S3 (C), and S4 (E). (B, D, F) Corresponding relationship between ellipsoid density and stimulation charge magnitude, plotted alongside somatic recruitment for patterns S2 (B), S3 (D), and S4 (F). Ellipsoid density is defined as the number of activated somata normalized by ellipsoid volume.

**Supplementary Figure 2.** Charge-dependent changes in 80% capture ellipsoid geometry for patterns S2–S4. (A–C) Variation in ellipsoid semi-axis lengths (x, y, and z radii) as a function of stimulation charge magnitude for patterns S2 (A), S3 (B), and S4 (C), computed using the 80% capture threshold.

## 1.2 Supplementary Tables

**Supplementary Table 1.** Capture threshold sensitivity analysis of the relationship between activation volume overlap and behavioral discrimination. IoU values were recalculated for each behaviorally evaluated ICMS pattern pair using ellipsoid capture thresholds of 75%, 80%, 85%, and 90%. The corresponding exponential decay fit coefficient of determination (R²) and leave-one-out sensitivity analysis results are shown for each threshold. Across all capture thresholds evaluated, the relationship between IoU and behavioral discrimination remained highly consistent, supporting the robustness of the primary findings.

| **ICMS Pattern Pair** | **Centroid Distance (µm)** | **Average Discrimination Accuracy (% ± SEM)** | **IoU (%) at Various Capture Thresholds** | | | |
| --- | --- | --- | --- | --- | --- | --- |
|  |  |  | **75%** | **80%** | **85%** | **90%** |
| S1 vs. S5 | 1200 | 89.9 ± 1.6% | 0 | 0 | 0 | 0 |
| S1 vs. S4 | 800 | 93.1 ± 0.9% | 0 | 0 | 0 | 0 |
| S1 vs. S3 | 400 | 72.6 ± 2.5% | 0 | 0.07 | 0.41 | 0.98 |
| S1 vs. S2 | 200 | 52.6 ± 1.8% | 17.15 | 18.92 | 20.87 | 23.27 |
| S1 vs. S1 | 0 | 46.4 ± 2.1% | 100 | 100 | 100 | 100 |
| F1 vs. F4 | 375 | 88.0 ± 1.9% | 0 | 0 | 0 | 0 |
| F1 vs. F3 | 250 | 80.6 ± 3.0% | 0 | 0.64 | 1.72 | 7.34 |
| F1 vs. F2 | 125 | 61.6 ± 2.7% | 16.52 | 22.21 | 24.26 | 28.87 |
| F1 vs. F1 | 0 | 49.1 ± 1.9% | 100 | 100 | 100 | 100 |
| **R^2^** | | | 0.88 | 0.88 | 0.89 | 0.88 |
| **Leave-One-Out R^2^ Range** | | | 0.85-0.95 | 0.85-0.95 | 0.87-0.96 | 0.85-0.96 |
